# Children with trisomy 21 are a group at risk for severe COVID-19: Case reports from a COVID-19 treatment unit in Addis Ababa, Ethiopia

**DOI:** 10.3389/fped.2022.991142

**Published:** 2022-10-13

**Authors:** Tinsae Alemayehu, Danilo Buonsenso

**Affiliations:** ^1^American Medical Center, Addis Ababa, Ethiopia; ^2^St. Paul’s Hospital Millennium Medical College, Addis Ababa, Ethiopia; ^3^Department of Woman and Child Health and Public Health, Fondazione Policlinico Universitario A. Gemelli IRCCS, Rome, Italy; ^4^Centro di Salute Globale, Università Cattolica del Sacro Cuore, Rome, Italy

**Keywords:** COVID-19, children, Down syndrome, SARS CoV-2, low and middle income countries

## Abstract

Trisomy 21 (Down syndrome) is a chromosomal disorder associated with humoral and cellular immunologic impairments among other systemic manifestations. It occurs at a frequency of 1 in 750 live births. There are increasing reports of children with Down syndrome presenting signs of severe COVID-19. The literature from Africa on pediatric COVID-19 and risk stratification for severe disease is scant. A summary of the clinical features, complications, and treatment outcomes of three Ethiopian children with Trisomy 21 and severe COVID-19 along with a discussion of the correlation between trisomy 21 immunology and severe SARS CoV-2 infection is presented.

## Introduction

Children with trisomy 21 (Down syndrome) exhibit various forms of immune dysregulation like a decrease in the number of immune cells and adverse antibody responses (1). Since the advent of the COVID-19 pandemic, there have been a few studies reporting on the increased vulnerability of this group of the population to severe disease, hospitalization, and death due to SARS CoV-2 (2). Descriptions of children with Down syndrome developing severe COVID-19 at an increased frequency have also been reported as early as February 2020 from registries in Italy (3). Epidemiologic studies of black African children with Down syndrome and SARS CoV-2 infection are sparse.

## Study site

The study site was the American medical center (AMC), Addis Ababa, Ethiopia. It is a multi-specialty private medical center that offers out-patient and in-patient clinical care. It specializes in infectious diseases and travel medicine synchronizing clinical consultations with advanced clinical bacteriology diagnostics.

Since the advent of the COVID-19 outbreak in Ethiopia, AMC has served as the only private pediatric COVID-19 center in Addis Ababa city handling admissions for children with severe and critical diseases (there are few other public pediatric COVID-19 treatment units). While overall, more than 180 children with varying severity of COVID-19 infections were treated throughout the entirety of 2021, the admissions unit had given care to 11 children in its first year of operation (December 2020–December 2021). Of those 11 children admitted for severe COVID-19 or multi-inflammatory syndrome associated with COVID-19 (MIS-C), three children have trisomy 21. The presentations of these three children with trisomy 21 and severe COVID-19 are summarized below.

## Case presentations

### Case I

A 10-year- and 10-month-old boy fell sick with fever, vomiting, cough, fast breathing, sore throat, and fatigue simultaneously with his parents. His past medical history was unremarkable except for his known trisomy 21. At presentation, he was tachypneic at 32 breaths per minute with the saturation of oxygen in room air of 89%. His temperature was 36.6*^o^*C and his pulse rate was 96 beats per minute. His lab work-up showed leukopenia (3,030 cells/mm^3^), lymphopenia (810/mm^3^), a slightly elevated serum CRP (11.2 mg/L), and a positive COVID-19 PCR test (cycle threshold or Ct value undocumented). Blood culture was not drawn. His chest x-ray showed a right middle lobe round pneumonia suggestive of bacterial pneumonia superimposed on a severe COVID-19 infection. He received treatment with intravenous ceftriaxone 75 mg/kg/day in two divided doses for 5 days and oral azithromycin 10 mg/kg/day as a single daily dose for 5 days, intra-nasal oxygen at 1–2 L/min, and intravenous dexamethasone 0.15 mg/kg once daily and recovered without complications. He was discharged after 3 days of hospitalization. He had no lingering symptoms or signs upon a follow-up evaluation a month after discharge. Due to the lack of studies on anticoagulant use upon the time of his admission (early 2021), he received no prophylactic anticoagulants.

### Case II

**FIGURE 1:**
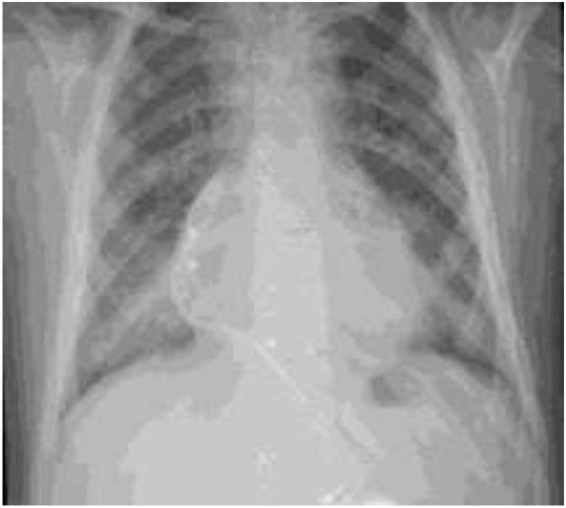
Chest x-ray of the second patient.

A 9-year- and 7-month-old boy presented with runny nose, cough, and vomiting 3 days prior to presentation. His past medical history was notable for his known trisomy 21, pacemaker implantation for a congenital arrhythmia, tympanostomy-tube insertion, and adenoidectomy for recurrent otitis media. He was not on any chronic medications. Upon physical examination, his pulse rate was 106 beats per minute, respiratory rate of 44 breaths per minute, saturation of oxygen in room air of 84%, and temperature of 37.5*^o^*C. Similar to the first child, he tested positive for COVID-19 PCR (Ct value: 34.2) with leukopenia for age (3,320 cells/mm^3^), lymphopenia (660/mm^3^), thrombocytopenia (88,000 cells/mm^3^), slightly elevated serum aspartate aminotransferase (AST) of 56 μ/L, elevated serum CRP (71 mg/L), elevated prothrombin time (PT) of 15.8 s and partial thromboplastin time (PTT) of 37.5 s. His blood cultures yielded no growth. His chest x-ray (Figure 1) showed peribronchial thickening and a right lower lobe consolidation. Following a 7-day treatment with intravenous ceftriaxone 75 mg/kg/day in two divided doses and oral azithromycin 10 mg/kg/day as a single daily dose for 5 days, subcutaneous enoxaparin 1 mg/kg in two divided doses and intravenous dexamethasone 0.15 mg/kg once daily for the duration of hospitalization, he recovered without complications. He was discharged after 8 days of stay in the hospital. Upon follow-up visits at 1 month and 4 months following discharge, he remained asymptomatic.

### Case III

A 1-year- and 2-month-old male toddler developed cough, grunting, fever, and few episodes of vomiting (blood streaked) for 2 days. His mother had tested positive for COVID-19 PCR a few days earlier with mild symptoms. He was under follow-up at a congenital cardiac illnesses’ clinic for patent ductus arteriosus and pulmonary hypertension and received daily oral furosemide and spironolactone. Upon admission, he was tachypneic at 50 breaths per minute with a saturation of oxygen in room air of 88%. His temperature was 38.5°C and his pulse rate was 134 beats per minute. He was underweight at 6.7 kg. He had fine crepitations over his right lower one-third lung.

**FIGURE 2:**
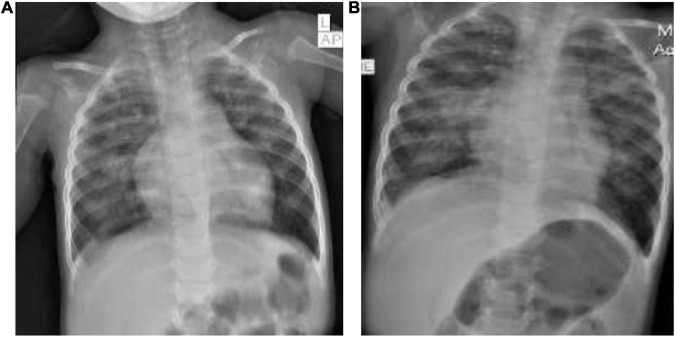
**(A,B)** Chest x-rays of the third patient.

He exhibited similar laboratory abnormalities as the previous two children: leukopenia for age (4,970 cells/mm^3^), lymphopenia for age (950/mm^3^), a slightly elevated serum CRP (12.9 mg/L), thrombocytopenia (119,000 cells/mm^3^), elevated serum aspartate aminotransferase (AST) of 106 μ/L, elevated prothrombin time (PT) of 13.2 s and partial thromboplastin time (PTT) of 42.1 s, and a positive COVID-19 PCR test (Ct value: 25.4). His initial chest x-ray (Figure 2A) showed abnormal cardiac shape with enlarged pulmonary vasculature, bilateral symmetrical perihilar, and paracardiac opacities.

After 5 days of intravenous ceftriaxone 75 mg/kg/day in two divided doses, oral azithromycin 10 mg/kg/day, subcutaneous enoxaparin 1 mg/kg/day in two divided doses, and intravenous dexamethasone 0.15 mg/kg/day as a single dose, his respiratory condition had not improved. His blood cultures revealed no growth and a repeat chest x-ray (Figure 2B) showed a right focal paracardiac opacity, left upper lobe patchy opacities, and right-sided minimal pleural effusion (hospital-onset superimposed pneumonia). His antibiotics were revised to intravenous vancomycin 60 mg/kg/day into four divided doses and cefepime 100 mg/kg/day into two divided doses which were given for 7 days. He required intermittent delivery of 0.5 L of oxygen per minute upon discharge following a 12-day hospitalization.

## Discussion

In this report, we describe three Ethiopian children with Down syndrome that developed severe COVID-19. To our knowledge, there are few descriptions of severe COVID-19 infection among children with trisomy 21 in low-income settings where there are limited options for diagnostics and therapeutics and courses may be complicated, while there have been some reports from mainly European countries (1). Overall, we found that the outcomes in the study setting were good based on our management protocol for children with severe COVID-19 comprising of oxygen supplementation, corticosteroids, anticoagulants, and antibiotics for suspected or documented superimposed bacterial pneumonia (2). While children with severe COVID-19 in sub-Saharan countries encounter higher mortalities of 8%, all patients in this case series survived without sequelae in accordance with many global pediatric cohorts with severe COVID-19 (4–7).

It is important to note that children who have a congenital or acquired immune compromise, infancy, comorbid conditions, chronic lung disease, obesity, and prematurity are at increased risk for severe COVID-19 (8–10). Keeping in mind that lymphopenia is one of the immune abnormalities associated with more than 90% of apparently healthy children with trisomy 21, its presence at the time of admission has also been shown to indicate poor prognosis in children with COVID-19 infection in terms of severity of infections, mortality, occurrence of acute respiratory distress syndrome (ARDS), and intensive care (ICU) admissions (11, 12).

Individuals with Down syndrome are at an increased risk for respiratory infections due to low T cell counts and serum immunoglobulin levels, decreased function of natural killer (NK) cells, elevated interferon (IFN) signaling, and altered toll-like receptor (TLR) signaling (13). Down syndrome has been cited as an independent risk factor for thrombosis in children (14).

Autopsy studies of pediatric deaths from COVID-19 inform us that the major underlying pathologies include vasculitis, fibrin-platelet thrombi notably in the lungs and heart, diffuse inflammatory alveolar damage, type II pneumocyte proliferation, hyaline membrane formation, hepatic necrosis and steatosis, pancarditis, etc. (15, 16). Half of a small number of autopsy studies in south African children who died from COVID-19 show bacterial superinfection (16). The increased predisposition of children with Trisomy 21 for thromboembolic events and qualitative and quantitative immune dysfunction thus predisposes for severe COVID-19 infection promoted by the aforementioned pathogenetic mechanisms.

Two of the three children with Down syndrome described in this report had congenital cardiopulmonary (patent ductus arteriosus and pulmonary hypertension in case 3, congenital arrhythmia in case 2) and nutritional (underweight in case 3) comorbidities which placed them on additionally higher risk for severe COVID-19 infection. All were males. Though male gender was reported to be a risk factor for severe SARS CoV-2 infection among adult patients, that wasn’t reciprocated among pediatric studies, but not among pediatric patients (17, 18). All three had varying degrees of leukopenia and lymphopenia.

Severe COVID-19 infection may complicate with organ dysfunction, venous thromboembolism and to a lesser extent with superinfections (19). Two of the three patients discussed developed superimposed pneumonia with no other systemic complications diagnosed during and in the immediate recovery periods. All three recovered from their illnesses. While more studies are being conducted to understand better the risk and prophylaxis for venous thromboembolism among children with COVID-19, available evidence shows that thrombophylaxis should be administered especially for those with a higher thrombotic risk like Trisomy 21, ages 12 years and older, children with malignancies as well as for those with multi-inflammatory syndrome associated with COVID-19 (MIS-C) (20–22).

In conclusion, we describe three Ethiopian children with trisomy 21 and severe COVID-19 and outline the correlation of the two syndromes. Our report provides so far rarely described details of an African pediatric cohort with Down syndrome presenting with severe COVID-19. As children with severe COVID-19 in low- and middle-income severe COVID-19. As children with severe COVID-19 in low- and middle-income practitioners should be alert to the immunologic risks this patient population carries.

Our report is limited by describing a single-center experience and by not accounting for a large number of patients. Further analysis of presentation and outcome of treatment for COVID among children with trisomy 21 is essential for a better understanding of the correlations, devise risk-stratified management and health advocacy.

## Data availability statement

The original contributions presented in the study are included in the article/supplementary material, further inquiries can be directed to the corresponding author.

## Author contributions

TA: conception and design of study, data collection, data analysis, and manuscript preparation and revision. DB: data analysis and manuscript preparation and revision. Both authors contributed to the article and approved the submitted version.
